# The long and winding road to causality

**DOI:** 10.1007/s10654-019-00507-4

**Published:** 2019-03-18

**Authors:** Olaf M. Dekkers

**Affiliations:** 1 Department of Clinical Epidemiology, Leiden University Medical Center, Leiden, The Netherlands; 2 Department of Clinical Epidemiology, Aarhus University Hospital, Aarhus, Denmark

Epidemiologists face two fundamental and interrelated problems when judging causality: knowledge is fallible, and studies are imperfect. In medicine, this will always leave a degree of uncertainty in scientific judgements. From an epistemological point of view, even randomized trials cannot be regarded as the ultimate proof to establish a causal relation. Given this inherent uncertainty it is no surprise that much attention has been drawn to the question how we can move from an association to a valid judgement of causation. It was exactly this question that urged Austin Bradford Hill more than 50 years ago to his well-known and still worth-reading paper, in which his nine viewpoints (often referred to as Hill’s criteria) to judge causality were described [1].

In their paper, published in this issue of the *European Journal of Epidemiology*, Olsen and Jensen call it a time for revision of Hill’s criteria [2]. They point towards the broad notion of new methodological developments in epidemiology that bring about the need for such a revision. No empirical argument is provided, in a sense that is assessed when and why these criteria were unable to display their role. Olsen and Jensen specifically argue for adding a consequence criterion to Hill’s list, ‘for epidemiological practice to be of use in real life’.

Very likely, Hill would have welcomed such discussion about his viewpoints; nowhere in his paper he claims that the presented list to judge causality is final, complete or sufficient. The only condition from Hill’s list that can be regarded as necessary, is temporality, as in medicine causes precede their consequences; however, as a feature to distinguish a mere association from causality, temporality is hardly helpful. Though not included as criterion, the fundamental prerequisite before judging causality is the presence of an association. This fundamental point is mostly disregarded, as the presence of an association is often the starting point for a discussion about causality; it can however be relevant to consider it for refutation of alleged causes, especially in pseudo-scientific debates where seemingly all meanings are equally important. In short: no causation without association.

None of the nine criteria is sufficient to judge causality and there is arguably also not a sufficient set or minimum number of Hill’s criteria that suffices for a verdict of causality. Not all criteria are equally important; for example, strength of the association and consistency are more relevant than specificity. How the different criteria have to be weighted in a specific case is also unclear. This issue is further complicated by the fact that many of the criteria are not independent. For example, the existence of experimental evidence (a stand-alone criterion), will influence the judgement of another criterion, plausibility. It is thus no surprise that in an empirical study, asking epidemiologists to make explicit reasons for the causality judgment for a specific exposure-outcome association, variation in the reasons, but also in the final judgment, was shown [3].

A major contribution to the field of epidemiology since Hill, is the introduction of a counterfactual framework to judge causality of interventions [4]. This framework, mentioned by Olsen and Jensen, has improved the field by further clarifying the structure of confounding and selection bias. An interesting question would be how Hill’s criteria relate to this counterfactual framework, which explicitly aims to frame the debate on causality of interventions. There is thus clearly room for a renewed discussion about the use(fulness) of Hill’s criteria, also as the scope of available data(bases) has changed considerably since then [5]. Whether it has merits to include a consequence criterion is less clear; let me argue by example.

In 2015, a Zika epidemic emerged and scientists aimed to assess whether causality was underlying the association of the Zika virus and microcephalia. Upfront, there were arguments in favour (association of a rare exposure with a rare outcome), but also against (no flavovirus had ever been shown to cause birth defects). As no single convincing piece of evidence existed, a group of experts evaluated existing evidence, thereby explicitly using two frameworks for causal inference, one of which Hill’s criteria [6]. The expert’s argued that most of Hill’s criteria were met, with the exception of two: there were no experimental data supporting the causal claim, and no data existed to judge whether a higher viral load translated into a higher risk for birth defects (dose–response relation). They finally, based on different lines of evidence, concluded that Zika was causing birth defects.

As can be seen from the Zika example, applying Hill’s criteria is not an automatic process that generates a correct conclusion; judging causality ultimately requires a verdict that has some degree of uncertainty. The fact that two sets of criteria were used (Hill’s and Shepard’s), does not add largely to the conclusion, as these criteria were partly overlapping. It is unlikely that a counterfactual framework of causality would have been better equipped in this case. Of course, one can think of Zika infection in terms of a hypothetical and unbiassed trial [7]. But even if such a hypothetical trial would have been defined, it is unlikely that the data would have matched the requirements of such hypothetical trial. For example, we might not know the precise timing of infection in most women, and women will likely differ with regard to duration of infection and viral load. Actually, the epidemiologic evidence from analytic studies was rather poor [8].

No experiments, no (emulated) trials, different lines of evidence and a question that was in urgent need for an answer. In such situation Hill’s framework is likely helpful to judge causality. Mind that the question at stake is broad: is the association between Zika and birth defects causal? For its answer different lines of evidence were combined and weighted. Such an approach, taking into account the total body of evidence, is more recently framed as triangulation [9]. After the verdict in the Zika case, further research was deemed necessary to answer relevant questions regarding the magnitude of the risk, the mediating factors of the risk or the effectiveness of potential therapeutic measures.

Let me turn to the proposal from Olsen and Jensen, that is adding a consequence criterion (what action can or should be taken, knowing that the association is causal) to Hill’s list. It was upfront clear in the Zika case, that a verdict of causality would have provided the basis for immediate discussion on public health action(s) to be taken. The potential need for action might even have contributed to the urge of the discussion about the causality. However, the potential for action, did not play a role in the ultimate verdict. Epistemologically, there is no compelling reason to argue that knowing the consequence (in terms of actions) of a casual judgment adds to the knowledge base of causality. It might even be that when judging causality, no certainty about relevant therapeutic options exists. Should we in such case abstain from a verdict of causality?

Hill was well aware of the relevance of actions, to which the last few paragraphs in his paper are devoted, as in passing from association to causation ‘we have to consider what flows from that decision’ [1]. He further argued that different levels of certainty in the causal verdict may be needed, depending on the action to be taken. For example, vaccination of an entire population requires strong evidence, whereas restricting the use of a specific drug in pregnancy for nausea because of the potential of a teratogenic effect, may be proposed based on weaker evidence. Thus, actions and consequences should in no way be neglected and they may even help to prioritize the agenda of medical research [10]. In this sense I agree with Olsen and Jensen that discussing consequences of epidemiological findings is crucial.

For therapeutic interventions, especially when considering implementation, a general statement about causality is not sufficient. Discussions about interventions require a clear definition of an intervention, and also the magnitude of the effect should be quantified. For causal inference of interventions, the counterfactual framework is clearly better equipped than Hill’s criteria. However, such counterfactual framework does not give us candidate exposures to be used as intervention, nor adds fundamentally to our understanding of why an intervention actually works. Before considering a substance or public health action as an intervention that is worthy of a RCT, this action should be considered as potentially disease reducing. How? By using the whole body of evidence about the condition, or the target, on which this action should work.

So, there is indeed room for discussing relevance and limitations of Hill’s criteria. And, if we are reconsidering criteria for causality anyway, I would like to add another urging challenge to the agenda: how to judge causality when data, analyses and probably even conclusions are provided by artificial intelligence.
